# Inhibition of DNA damage repair by artificial activation of PARP with siDNA

**DOI:** 10.1093/nar/gkt522

**Published:** 2013-06-12

**Authors:** Amelie Croset, Fabrice P. Cordelières, Nathalie Berthault, Cyril Buhler, Jian-Sheng Sun, Maria Quanz, Marie Dutreix

**Affiliations:** ^1^Institut Curie, CNRS-UMR3347, INSERM-U1021, 91405 Orsay, France, ^2^DNA Therapeutics, Génopole, 91000 Evry, France, ^3^Institut Curie, CNRS-UMR3348, Plateforme PICT-IBiSA, 91405 Orsay, France and ^4^Museum National d’Histoire Naturelle, USM503, 75231 Paris, France

## Abstract

One of the major early steps of repair is the recruitment of repair proteins at the damage site, and this is coordinated by a cascade of modifications controlled by phosphatidylinositol 3-kinase-related kinases and/or poly (ADP-ribose) polymerase (PARP). We used short interfering DNA molecules mimicking double-strand breaks (called Dbait) or single-strand breaks (called Pbait) to promote DNA-dependent protein kinase (DNA-PK) and PARP activation. Dbait bound and induced both PARP and DNA-PK activities, whereas Pbait acts only on PARP. Therefore, comparative study of the two molecules allows analysis of the respective roles of the two signaling pathways: both recruit proteins involved in single-strand break repair (PARP, XRCC1 and PCNA) and prevent their recruitment at chromosomal damage. Dbait, but not Pbait, also inhibits recruitment of proteins involved in double-strand break repair (53BP1, NBS1, RAD51 and DNA-PK). By these ways, Pbait and Dbait disorganize DNA repair, thereby sensitizing cells to various treatments. Single-strand breaks repair inhibition depends on direct trapping of the main proteins on both molecules. Double-strand breaks repair inhibition may be indirect, resulting from the phosphorylation of double-strand breaks repair proteins and chromatin targets by activated DNA-PK. The DNA repair inhibition by both molecules is confirmed by their synthetic lethality with BRCA mutations.

## INTRODUCTION

To overcome DNA damage, cells have evolved mechanisms to detect DNA lesions, signal their presence and promote their repair (1–3). The wide diversity of types of DNA lesion necessitates multiple and generally independent DNA repair mechanisms. Although responses to different classes of DNA lesions differ, most occur via signal transduction cascades involving post-translational modifications, such as ubiquitination, phosphorylation, acetylation and poly (ADP-ribosy)lation (PAR also called PARylation). Key regulators within these signaling cascades, such as the phosphatidylinositol 3-kinase-related kinases (PI3K) Ataxia Telangiectasia Mutated (ATM), Ataxia Telangiectasia and Rad3-related (ATR) or DNA-dependent protein kinase (DNA-PK) and the poly [Adenosine Diphosphate (ADP)-ribose] polymerase (PARP), are activated via direct or indirect interaction with double-strand breaks (DSB) and single-strand breaks (SSB) (4–6).

The DNA damage most toxic to the cell is DSB, which, if left unrepaired, leads to loss of chromosome fragments and cell death. Cells have two major pathways to repair DSB: homologous recombination (HR) and non-homologous end-joining (NHEJ) (7,8). These pathways are complementary and operate optimally during the S and G_2_ phases of the cell cycle for HR (9) and throughout all cell cycle for NHEJ pathway (10–12). Thus, during S and G_2_ phases of the cell cycle, DSB are preferentially repaired by HR between sister chromatins. An important regulatory step that determines the choice between the NHEJ and HR pathways is the process of DSB by the MRE11-RAD50-NBS1 complex (MRN), in conjunction with other factors. After resection of DSB ends, the resulting single-strand DNA ends are coated with replication protein A (RPA) and then RAD51 with the help of RAD52, breast cancer 2 (BRCA2) and Fanconi anemia (FANC) proteins; these proteins promote invasion and strand exchange with the homologous region on the sister chromatin. Thereafter, repair proceeds either via the double Holliday junction model DSB repair pathway or via the synthesis-dependent strand-annealing pathway. In mammalian cells, NHEJ is the major pathway for repairing breaks not associated with replication. NHEJ involves the direct rejoining of two damaged DNA ends in a sequence-independent manner (13,14): DNA ends are first bound by the Ku70/Ku80 heterodimer, which recruits and activates the catalytic subunit, DNA-PKcs, to form the DNA-PK holoenzyme (15). Broken DNA ends are then processed and ligated by a set of enzymes, including Artemis, polynucleotide kinase, X-ray cross-complementing 4 (XRCC4) and ligase IV. If the classical mechanism of NHEJ is impeded, an alternative end-joining pathway operates that involves factors of HR and SSB repair, including MRN complex, PARP-1, XRCC1 and DNA ligase I or III (4).

Although less harmful than DSB, SSB are toxic. One of the most common sources of SSB is oxidative attack by endogenous reactive oxygen species. In the case of free radicals from hydrogen peroxide (H_2_O_2_), a physiologically relevant source of reactive oxygen species, SSB occurs three orders of magnitude more frequently than DSB (16). After exposure to ionizing radiation, SSB are 25 times more abundant than DSB (17). They are primarily detected by PARP-1, although other members of the PARP super family may contribute (18–20). Binding of PARP to SSB triggers poly (ADP-ribosy)lation of numerous nuclear proteins including PARP. These modifications in turn promote the binding of XRCC1, which acts as a molecular scaffold for SSB repair components (21). Therefore, PARP, which binds to DSB with a greater affinity than that for its binding to SSB (22), is involved both in repair of both, whereas the recruitment of DNA-PK by Ku is strictly dependent on DSB and seems to be involved in DSB repair only.

The outcome of DNA damage signaling is, literally, a matter of life or death. Depending on the severity of the DNA damage, the cell will either repair the damage to enable it to continue dividing, or enter apoptosis. Our understanding of the dynamics of the repair proteins has been greatly advanced through the use of various types of DNA substrates in biochemical assays. We recently showed that short interfering double-stranded DNA molecules (siDNA) that mimic DSB damage (called Dbait) induce a partial damage response in cells and can be used to analyze the early steps of repair protein recruitment *in vivo* (23). Dbait molecules activate DNA-PK kinase and have no significant effect on other PI3K kinases (24). In the course of this response, several nuclear DNA-PK targets, such as p53, RPA32 or H2AX, are extensively phosphorylated. The activation of DNA-PK prevents recognition of further DSB and inhibits not only the NHEJ, which directly depends on DNA-PK, but also HR pathways. In this work, we investigate the ability of Dbait to activate the PARP enzyme and the effects on SSB repair protein recruitment and activity.

To distinguish between DNA-PK activation and PARP activation, we designed new siDNA molecules, called Pbait, that mimic SSB and specifically activate PARP polymerase. Both Dbait and Pbait molecules induced PARP activation and inhibit its repair activity by trapping most of the proteins involved in SSBR.

## MATERIALS AND METHODS

### Cell culture, Dbait molecules and treatments

**Figure 1.**
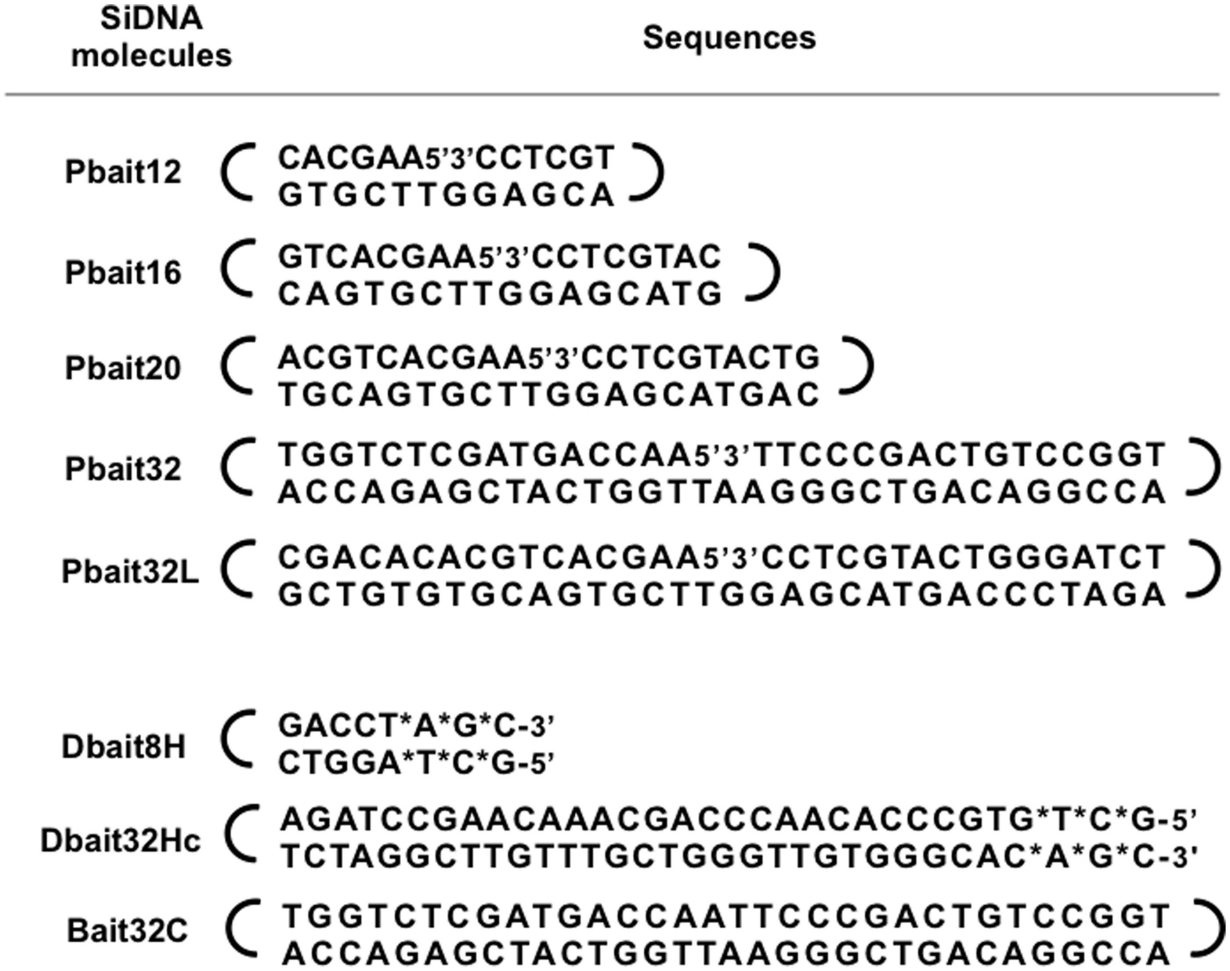
siDNA molecule sequences. Dbait molecules are linear duplex DNAs with one hexaethyleneglycol linker (gray bracket) at one end and three phosphorotioate modifications (asterisk) at the other end. Pbait molecules are linear duplex DNAs with an interruption in the middle of one strand (noted 5'3') and a hexaethyleneglycol linker at each end. Bait32C is a linear duplex DNA with a hexaethyleneglycol linker at each end.

The cell lines used in this study were SV40-transformed MRC-5 (ATCC number CCL-171), HeLa (ATCC number CCL-2), DNA-PK-defective M059J (ATCC number CRL-2366), M059K (ATCC number CRL-2365), MDA-MB-231 (ATCC number HTB-26), HCC1937 (ATCC number CRL-2336) and HeLa Silencix cells (Tebu-Bio number 00301-00041 for BRCA1 HeLa Silencix and number 00301-00028 for BRCA2 HeLa Silencix). Cells were grown according to the supplier’s instructions (ATCC, Molsheim, France and Tebu-Bio, Le-Perray-en-Yvelines, France). siDNA molecules (Figure 1) were obtained by automated solid-phase oligonucleotide synthesis from Eurogentec (Seraing, Belgium). Cultures were transfected with siDNA molecules with jetPEI (Polyplus-transfection, Illkirch, France) at an N/P ratio of 6 according to the manufacturer’s instructions. Unless otherwise indicated, cells were transfected at 80% confluence, with 2 μg of siDNA in 1.3 ml of culture medium (0.1 μM) without Fetal Calf Serum (FCS) (in 60-mm-diameter plates) for 5 h. Hydrogen peroxide solution (H_2_O_2_; Sigma-Aldrich, St Louis, USA) was used as a DNA damaging agent. PARP inhibitor ABT-888 was purchased to Selleckchem (Euromedex, Souffelweyersheim, France).

### Plasmids and transfection

The plasmid XRCC1-EYFP (Enhanced Yellow Fluorescent Protein) was a gift from P. Radiccella (DSV, CEA, Fontenay-aux-roses, France). Cells were transfected with XRCC1-EYFP (2 μg) in Superfect reagent (20 μl; Qiagen, Courtaboeuf, France) in 1.2 ml of medium (in 60-mm-diameter plates) for 5 h, and then grown to express recombinant protein for at least 48 h.

### Antibodies and immunological techniques

The following antibodies were used: polyclonal rabbit anti-poly (ADP-ribose), purified mouse anti-poly (ADP-ribose) (BD Pharmigen, Le Pont de Claix, France), monoclonal mouse anti-PARP C2-10 (Trevigen, Gaithersburg, USA), polyclonal rabbit anti-PCNA (Cell Signaling, Boston, USA), monoclonal mouse anti-DNA-PK (NeoMarker, Fremont, USA), polyclonal rabbit anti-Ku70 (Santa Cruz Biotechnologie, Heidelberg, Germany), polyclonal rabbit anti-NBS1 (Novus Biologicals, Cambridge, UK), polyclonal rabbit anti-MRE11 (Novus Biologicals, Cambridge, UK), polyclonal rabbit anti-phospho H2AX (Millipore, Billerica, France) and monoclonal mouse anti-phospho-H2AX (Millipore, Billerica, France). For immunostaining, cells grown on cover slips (Menzel, Braunschweig, Germany) were fixed for 20 min in 4% formaldehyde/phosphate-buffered saline (PBS) 1×, permeabilized in 0.5% Triton X-100 for 10 min, blocked with 2% bovine serum albumin/PBS 1× (or 2% non-fat milk/PBS 1×) and incubated with primary antibody for 1 h at room temperature (RT) or overnight at 4°C. All secondary antibodies conjugated with Alexa-488 or Alexa-633 (Molecular Probes, Eugene, OR, USA) were used at a dilution of 1/200 for 30 min at RT, and DNA was stained with 4',6-diamidino-2-phenylindole (DAPI). For immunoblotting, cells were boiled in sodium dodecyl sulfate sample buffer (50 mM Tris–HCl, pH 6.8, 1% β-mercaptoethanol, 2% sodium dodecyl sulfate, 0.1% bromophenol blue and 10% glycerol). Proteins were separated by electrophoresis in 5 or 12% acrylamide/bisacrylamide (35.5/1) gels, transferred to nitrocellulose membranes, blocked with Odyssey buffer for 1 h and hybridized overnight at 4°C with primary antibody. Blots were then incubated with IRD secondary antibodies at 1/10 000 dilution (A700 or A800), and protein–antibody complexes were revealed on Odyssey (LI-COR Biotechnology, Bad Homburg, Germany).

### PARP and DNA-PK activity assay

DNA-PK and PARP activities were monitored using the SignaTECT DNA-dependent Protein Kinase Assay System kit (Promega, Madison, USA) and Universal Chemiluminescent PARP Assay Kit with Histone-Coated Strip Wells (Trevigen, number 4676-096-K, Gaithersburg, USA). Phosphorylation reactions were performed with 50 U of DNA-PK and 0.25 μg (500 nM) of siDNA, or 1.5 μg of diethylaminoethyl cellulose (DEAE)-purified MRC5 cell extract and 0.01 μg (20 nM) of siDNA. The ribosylation reaction included 0.10 μg (200 nM) of siDNA and 0.5 U/well PARP-HSA or 40 μg of MRC5 cell extract. Negative controls were Dbait8H and Bait32C.

### Trypan blue survival test

Cells were seeded in six-well plates at concentration of 10^5^ cells per well. Triplicate wells were processed for each experimental point. Treatments were performed the day following seeding. Cells were then allowed to grow for 1 or 24 h after treatment, treated with 0.025% trypsin and stained with 0.4% trypan blue (Sigma Aldrich, Saint-Louis, USA). Cells were counted under microscope using a Bürker counting chamber. Survival is estimated as a percentage of blue cells on the total number of cells.

### Single-cell gel electrophoresis comet assay

Cells transfected or not transfected with siDNA were analyzed for DNA damage by an alkaline ‘comet assay’ as described in Quanz *et al.* (24). Duplicate slides were processed for each experimental point. The tail moment is defined as the product of the percentage of DNA in the tail and the displacement between the head and the tail of the comet (25).

### Inducing photo-damage

These experiments were performed with a Leica SP5 confocal system, attached to a DMI6000 stand using a ×63/1.4 objective, under a controlled environment (37°C, 5% CO_2_). All recordings were made using the appropriate sampling frequency (512 × 512 images, line average of four and zooming set to eight) and an argon laser line (514 nm for YFP) adapted to the fluorescent protein of interest. In the first step, two images were acquired within a period of 2–3 s at a laser energy setting sufficiently low not to induce any photodynamic damage. The 405-nm laser line (diode) was then set to maximum output for 100 ms and focused onto a single spot of constant size (176 nm) within the nucleus to cause a point of photo damage with a reproducible amount of energy. Recruitment of the protein of interest was then monitored by fluorescence using the same setting as for the pre-damage sequence. Laser damage was induced 6 h after the beginning of siDNA transfection. Images were captured at 2–5 s intervals for the following 70 s (26).

## RESULTS

### Design of siDNA molecules mimicking SSB

The siDNA used was short double-strand DNA that carries a single-model lesion. Dbait molecules, that mimic DSB, were constructed by tethering two complementary oligonucleotides with an hexaethyleneglycol linker at one extremity of the duplex and protecting the ends at the other extremity by adding three phosphorotioate modifications (Figure 1) (23,24). We designed new siDNA molecules mimicking SSB (called Pbait), these constructs form a duplex with no free end and an interruption in the middle of one strand (Figure 1). Each end of the Pbait molecules was tethered by a hexaethyleneglycol loop to prevent DNA-PK binding (Figure 1). The duplex length in Pbait molecules was between 8 and 32 bp (Figure 1).

**Figure 2.**
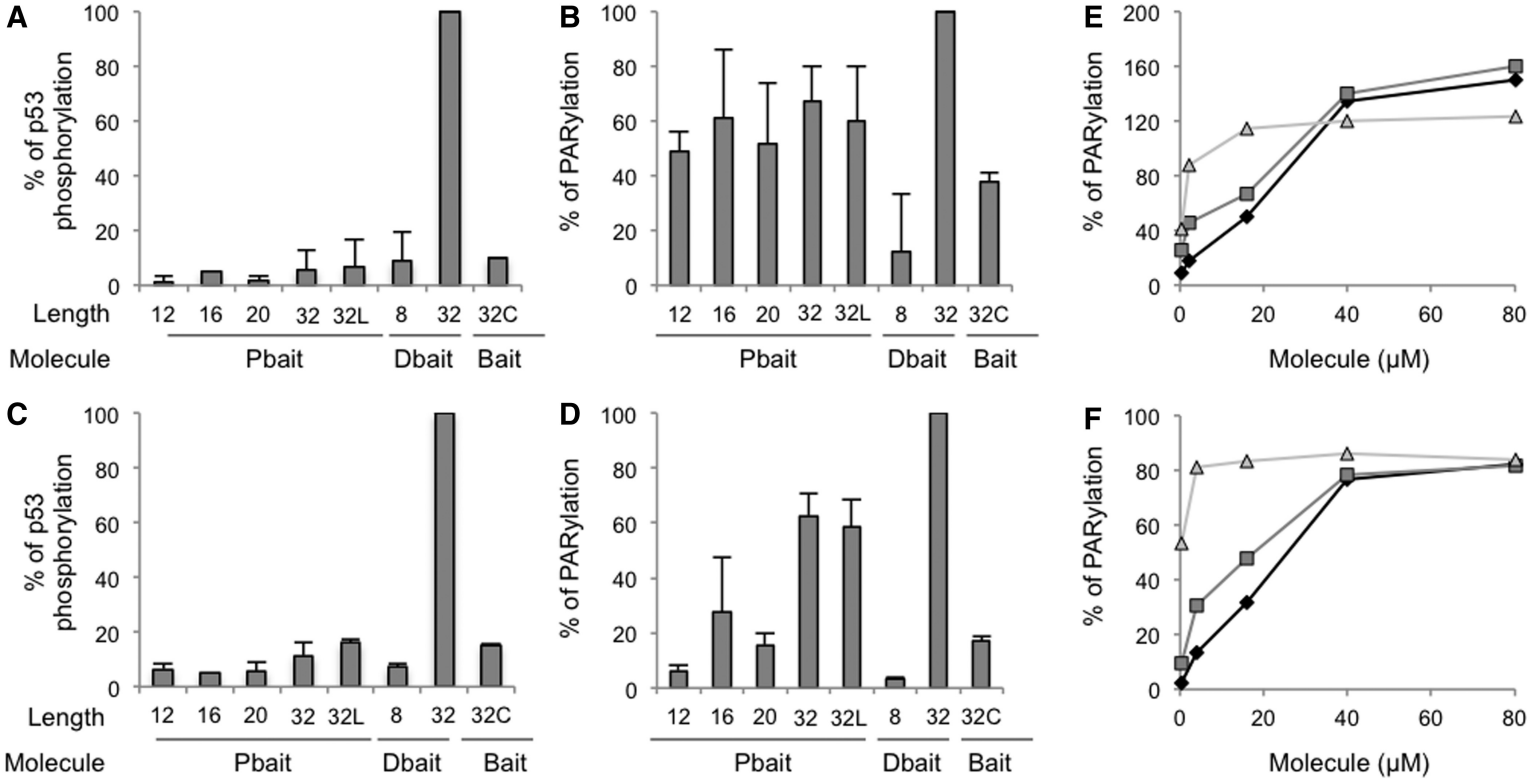
PARP and DNA-PK activation induced by siDNA. PARylation and DNA-PK kinase activities were measured either with purified enzymes (**A, B** and **E**) or nuclear extracts from MRC5 cells (**C, D** and **F**): kinase activity was estimated by measuring P53 peptide phosphorylation after addition of 0.5 µM siDNA (A, C). PARP activation was estimated by measuring H1 histone parylation after addition of 0.2 µM siDNA (B, D) or increasing amounts of siDNA (Panel E, F: Pbait32, triangle; Pbait12, square; Dbait8H, diamond). Reported values represent the mean value and standard deviation of at least three independent experiments. Two 32-bp-long Pbait molecules with different sequences were tested (Pbait32 and Pbait32L). The Dbait8H and Bait32C were used as negative control.

siDNA molecules were screened for their ability to recruit PARP and induce the synthesis of a poly (ADP-ribose) chain (PAR) referred to as PARylation (Figure 2). PARP activity assays were performed using purified PARP enzyme (Figure 2B and E) and MRC5 cell extracts (Figure 2D and F). Dbait32Hc and Pbait32 molecules (32 bp) efficiently activated both purified PARP and PARP in crude extracts. These results confirm previous observations that PARP binds to DSB with a high affinity (22). Shorter Dbait or Pbait molecules did not efficiently activate PARP in crude extract. Analysis of PARP activation as a function of siDNA concentration showed that maximal activation required 10-fold more Pbait12 than Pbait32 (Figure 2E and F). As expected, Bait32C molecules that have no nick or free ends did not activate PARP. Pbait32L and Pbait32 that have the same structure but differ in DNA sequence (Figure 1) had similar PARP activation activities. PARP activation thus did not depend on the DNA sequence. Moreover, only Dbait and not Pbait activated DNA-PK whether as a purified enzyme (Figure 2A) or in MRC5 cell extracts (Figure 2C). This result is in agreement with previous observation that 34–32-bp long dumbbell form with no free ends does not bind DNA-PK (27). We chose the 32-bp-long Pbait and Dbait molecules (hereafter called Pbait32 and Dbait32Hc), which activated PARP and PARP/DNA-PK, respectively, for further studies in cell cultures. We used an 8-bp-long Dbait (Dbait8H), which does not activate DNA-PK or PARP, as a transfection control (Figure 2).

### Pbait and Dbait molecules induce PARylation in cells

**Figure 3.**
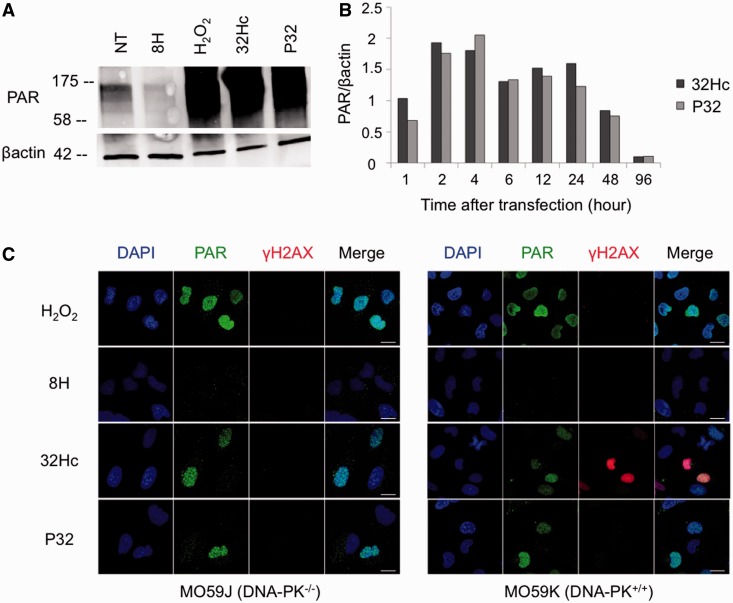
siDNA molecules induce PARylation. (**A**) Western blot of proteins from MRC5 cells treated with hydroxide peroxide (H_2_O_2_), Dbait8H, Dbait32Hc or Pbait32. Cell extracts were prepared 1 h after the end of transfection or 10 min after H_2_O_2_ treatment (500 μM). (**B**) Kinetics of PARylation induced by Pbait32 (light gray) and Dbait32Hc (dark gray) in MRC5 cells, 1–96 h after the end of transfection. (**C**) Immunofluorescence of PAR (green) and γH2AX (red) in MO59J (DNA-PK deficient) cells and MO59K (DNA-PK wild-type) cells transfected with Dbait8H, Dbait32Hc or Pbait32 molecules or treated with H_2_O_2_. Scale bar: 20 µM.

We confirmed PARP activation in cells by monitoring PAR-modified proteins in cells transfected with Dbait32Hc or Pbait32. Substantial PAR-modification of proteins was observed in cells after transfection with both siDNA (Figure 3A), consistent with the enzymatic assays. Short Pbait with size ranging, from 12 to 20 bp, or Bait32C with no nick did not induce protein PARylation in treated cells (Supplementary Figure S1). The extent of PARylation in cells with PARP activated by Dbait32Hc and Pbait32 was similar to that observed after oxidative stress induced by H_2_0_2_ treatment (Figure 3A). Kinetics of PARylation by the two siDNA were similar with an activity maximum 4 h after beginning of treatment (Figure 3B)*.* Nicotinamide adenine dinucleotide (NAD) consumption, as NAD is used as a substrate by PARP for PARylation of its target proteins, can monitor PARP activity in the cell. As expected, the NAD concentration decreased as the PAR signal increased in cells transfected with Pbait32 and Dbait32Hc, but not with the control Dbait8H (Supplementary Figure S2). The kinetics of NAD consumption coincided closely with the kinetics of PARP activation (Figure 3B).

**Figure 4.**
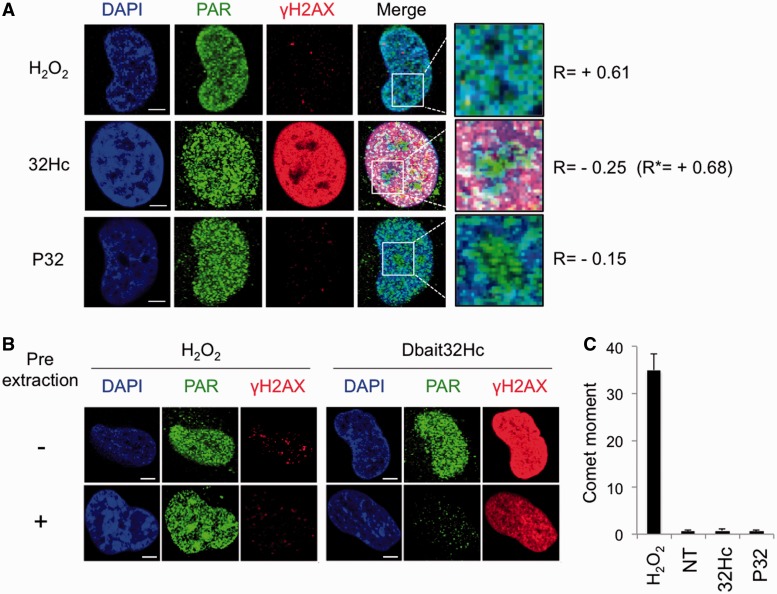
PARylation induced by siDNA is mostly not associated with chromosomes. (**A**) Immunodetection of PAR polymers (green) and γH2AX (red) in MRC5 cell lines after transfection with siDNA or H_2_O_2_ treatment. White squares are magnified 3.5 times. Pearson coefficients of co-localization between DAPI and PAR (R values) or DAPI and γH2AX (R* value) were determined with *ImageJ software*. (**B**) Immunofluorescence of PAR (green) and γH2AX (red) in MRC5 pre-treated (+) or not (−) with a pre-extraction buffer. (**C**) DNA damage after H_2_O_2_ or siDNA treatments analyzed by alkaline comet assay. The moment value reported is the mean value from three independent measurements of comet tails estimated from 100 nuclei per condition. Scale bar: 5 μM.

siDNA activated PARP in various tumor cell lines derived from melanoma, larynx and cervix (Supplementary Figure S3), glioblastoma (Figure 3C) and in MRC5-transformed fibroblasts (Figure 4). In agreement with the enzymatic assay results, only Dbait32Hc treatment induced DNA-PK activation, as revealed by testing for the phosphorylated form of the histone variant H2AX, called γH2AX (Figure 3C). H2AX phosphorylation but not PARylation activity was affected by the DNA-PK defect in the MO59J cell line (Figure 3C and Supplementary Figure S4). These results indicate that H2AX phosphorylation by Dbait is strictly dependent on DNA-PK, but that in contrast PARP activation does not require DNA-PK.

### Pbait and Dbait recruitment of DNA repair proteins

Foci of PAR modification were observed in nucleus of siDNA-treated cells. The localization of the foci induced by Dbait differed from those formed after H_2_0_2_ treatment to DNA damage. PAR foci induced by H_2_0_2_ perfectly co-localized with heterochromatin stained with DAPI, but those induced by Dbait32Hc and Pbait32 were distributed across the nuclei independently of chromatin condensation (Figure 4A). A pre-extraction treatment to remove soluble molecules before fixing the cells (Figure 4B) suppressed the PAR signal in Dbait-treated cells but not in H_2_O_2_-treated cells. This confirms that PAR modifications induced by siDNA are mainly on soluble molecule complexes rather on chromatin. Numerous damages were detected by alkaline comet assay on chromosomes after H_2_O_2_ treatment and probably correspond to the sites of PARP activation in the cell; no chromosome damage was detected after siDNA treatments (Figure 4C). Thus, PAR foci appear to form where the DNA damage is detected: on chromosomes after H_2_O_2_ and on Dbait32Hc or Pbait after siDNA treatments.

**Figure 5.**
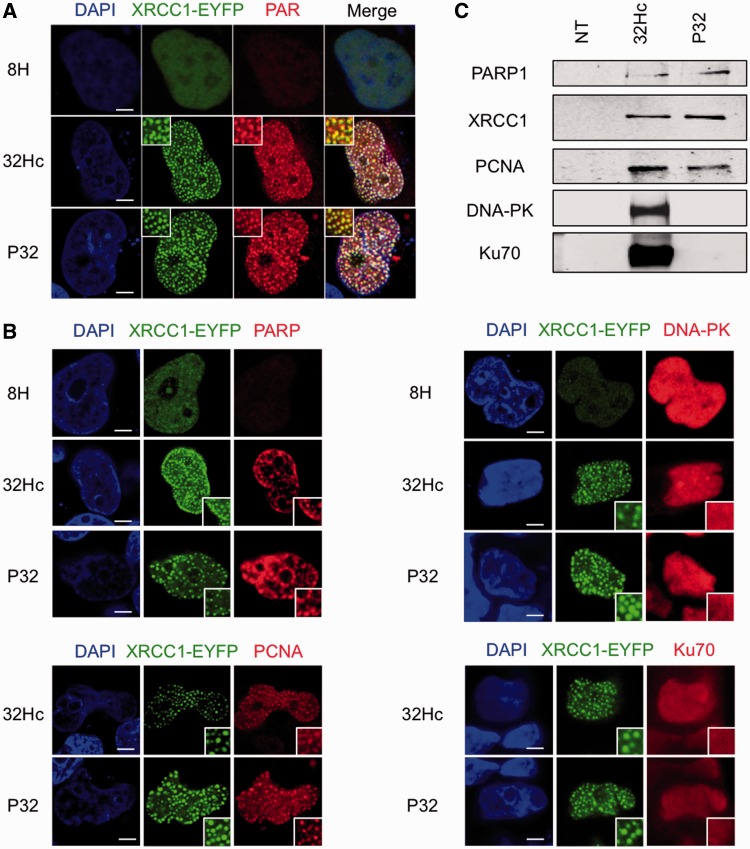
Proteins accumulation on siDNA. (**A**) Immunodetection of PAR and XRCC1-EYFP 6 h after beginning of siDNA transfection. (**B**) Immunodetection of PARP, PCNA, DNA-PK and Ku70 proteins and XRCC1-EYFP after siDNA transfection. (**C**) Pull-down assay with biotinylated siDNA. Western blotting reveals proteins trapped by biotinylated siDNA. Scale bar: 5 μM.

Self PARylation of PARP is an early event at SSB sites. XRCC1 is then rapidly recruited to the sites of PAR synthesis (28,29), whereas PCNA (proliferating cell nuclear antigen) is recruited more slowly (30,31). After transfection with siDNA, PARP, XRCC1 and PCNA accumulate in foci that co-localized (Figure 5). We first demonstrated that XRCC1 tagged with an EYFP peptide co-localized with PAR foci (Figure 5A) then used this same construct to demonstrate co-localization of both PARP and PCNA with EYFP-XRCC1 (Figure 5B). In contrast, DNA-PK and Ku did not form foci in Dbait32Hc-treated cells, although the activation of the kinase activity revealed their interaction with the DNA bait (Figure 5B). They show a uniform distribution in nucleus after siDNA treatment, indicating that their binding to Dbait32Hc did not lead to their aggregation in foci.

To confirm that all these repair proteins bind directly or indirectly to the siDNA, we performed pull-down assays with biotinylated Dbait and Pbait molecules. Cells were transformed with biotinylated siDNA, and proteins bound to these baits were retrieved on streptavidin beads. Both Pbait32 and Dbait32Hc recruited the SSB repair proteins (PARP, XRCC1 and PCNA), but only Dbait32Hc, and not Pbait32, recruited the NHEJ repair proteins such as DNA-PK and Ku70 (Figure 5C).

### siDNA inhibits XRCC1 and PCNA recruitment at damage sites

**Figure 6.**
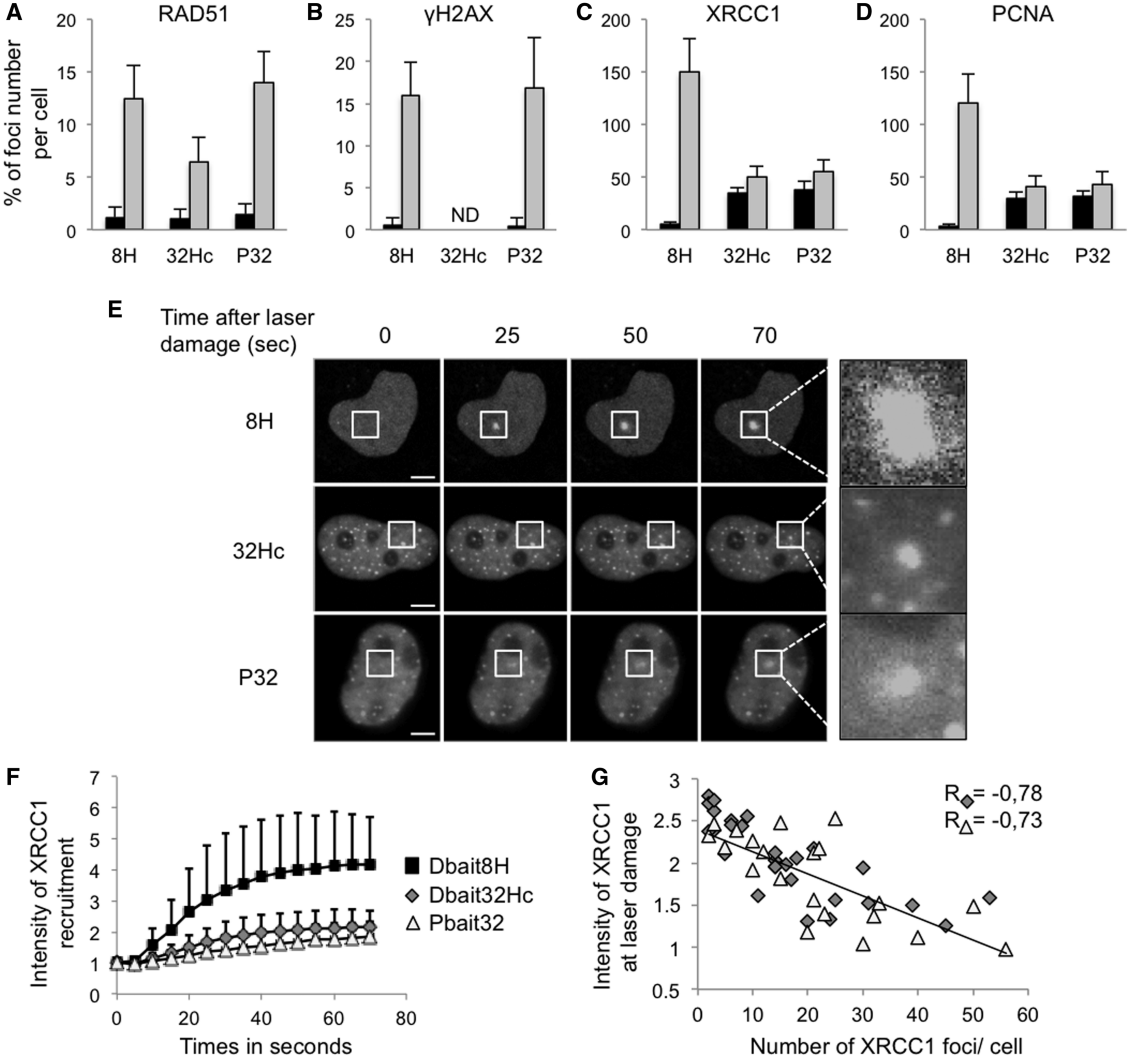
Pbait prevents the re-localization of SSB but not DSB repair proteins. Numbers of foci of RAD51 (**A**), XRCC1 (**B**) and PCNA (**C**) proteins formed in cells transfected with Pbait32, Dbait32Hc or Dbait8H and irradiated with 10 Gy (gray) or non-irradiated (black). (**D**) Distribution of γH2AX in 10 Gy irradiated cells with (gray) or without Pbait32 treatment (black). (**E**) Kinetics of XRCC1-EYFP re-localization to laser damage sites (white squares) after siDNA treatment. White squares are magnified 5.5 times; (**F** and **G**) microcopy quantifications of XRCC1-EYFP foci at various times after laser damage; (F) mean value of 100 laser-treated cells; (G) inverse correlation between numbers of XRCC1-EYFP foci in cells transfected with Pbait or Dbait at time zero and maximal recruitment at laser induced damage. Black square: Dbait8H; Gray diamonds: Dbait32Hc; White triangle: Pbait32. Scale bar: 5 μM.

We previously demonstrated that Dbait32Hc treatment prevents recruitment of DSB repair proteins, such as 53BP1, RAD51 and NBS1, at sites of damage induced by irradiation (23,24). This inhibition could be a consequence of the activation of PARP as well as DNA-PK signaling enzymes by Dbait. To determine the role of PARP activation in the recruitment of these DSB repair proteins, we analyzed the recruitment of the RAD51 protein at site of DNA damage induced by irradiation in siDNA-transfected cells. As previously shown, we found that Dbait significantly reduced recruitment of RAD51 (Figure 6A), NBS1 or MRE11 (Supplementary Figure S5) (23,24). In contrast, Pbait had no effect on RAD51, NBS1 and MRE11 recruitment, suggesting that the inhibition of DSB repair proteins by Dbait is probably specific to DNA-PK activation (23,24). In agreement with this result, we observed that the number of γH2AX foci induced by irradiation was similar in untreated cells and Pbait32-treated cells (Figure 6B). We investigated whether PARP activation by the siDNA inhibited recruitment of the proteins involved in SSB repair at damage sites. In the absence of siDNA treatment, 10 Gy irradiation induced ∼10-fold more XRCC1 and PCNA foci than RAD51 foci (Figure 6A, C and D); this reflects the greater number of SSB than DSB caused by irradiation. In contrast to RAD51, MRE11 or NBS1, the number of XRCC1 and PCNA foci did not increase after irradiation of cells transfected with Dbait32Hc or Pbait32 (Figure 6C and D), indicating that both siDNA prevent recruitment of SSB repair proteins to damage site on chromosomes.

To confirm this result, we followed the movements of EYFP-XRCC1 in real-time after laser-induced damage (Figure 6E and F). The rate of recruitment of EYFP-XRCC1 at sites of laser-induced damage was significantly lower in all cells treated with Dbait or Pbait than controls; the maximal amount of recruited proteins for siDNA-treated cells was half that for controls. The extent of inhibition as assessed from the amount of XRCC1 recruited in the 70 s after laser treatment directly correlated with the number of XRCC1 foci present before treatment. This indicates that as more XRCC1 protein was trapped in siDNA-induced foci, less XRCC1 protein was localized at damage sites (Figure 6G). These results show that the substantial damage caused by laser irradiation was not sufficient to displace proteins from siDNA to sites of chromosome damage.

### Dbait and Pbait are synthetic lethal with BRCA mutations

**Figure 7.**
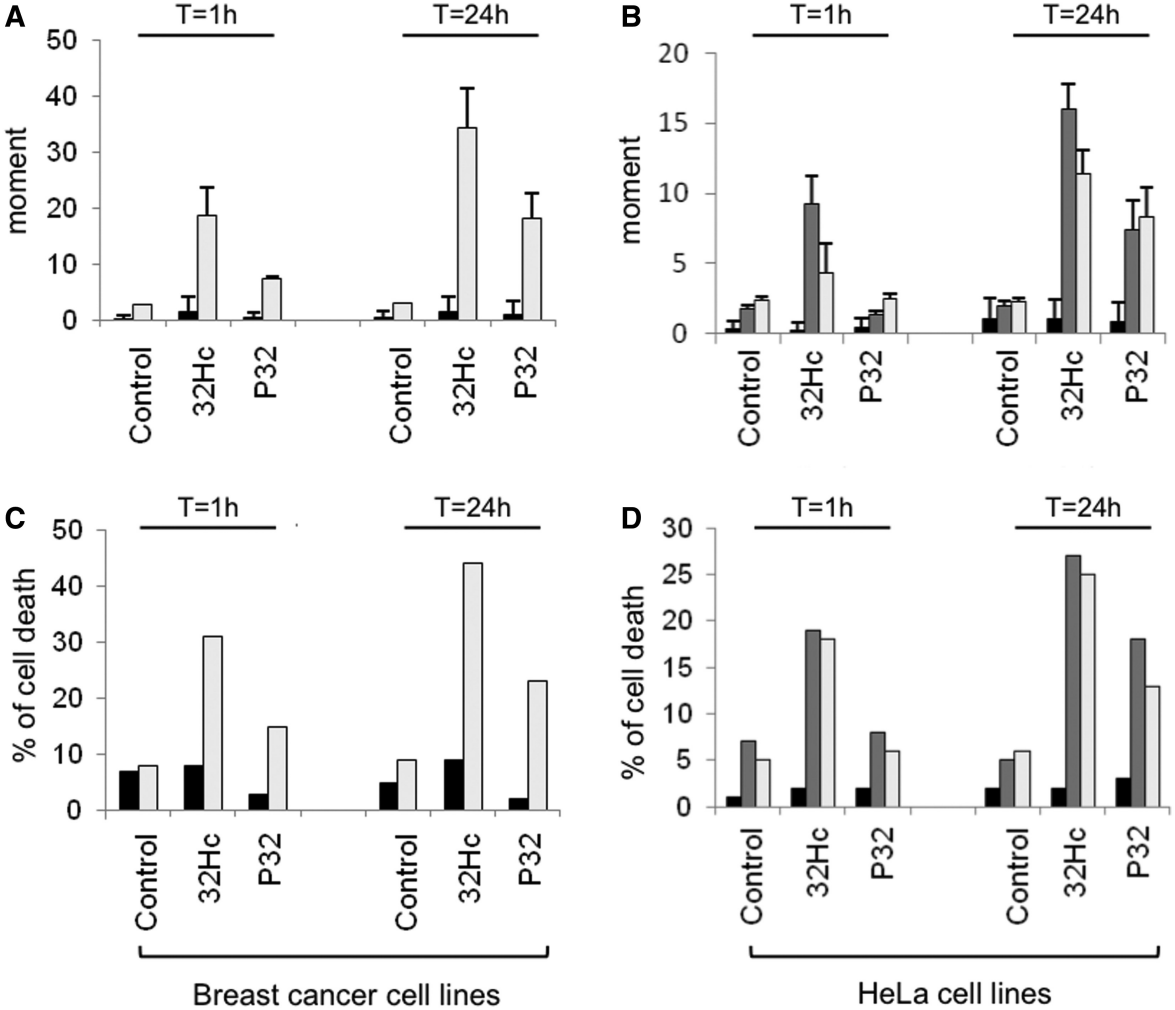
siDNA is synthetic lethal with BRCA mutations. DNA damage was monitored in Pbait32- or Dbait32Hc-treated cells by comet assay (**A** and **B**), and survival was estimated by Trypan blue cell counting (**C** and **D**). Analyses were performed in breast cancer cell lines [panels A and C: MDA-MB-231 (BRCA^+/+^), black; HCC1937 (BRCA1^−/−^), gray] and in HeLa cells (panels B and D: HeLa, black; HeLa_shBRCA1, dark gray; HeLa_shBRCA2, gray). Values are mean value of at least three independent experiments.

The inhibition of PARP, XRCC1 and PCNA foci formation at damage sites suggests that Dbait and Pbait, like PARP inhibitors, inhibit SSB repair. PARP inhibitors are lethal to cells that are already deficient in DSB repair but have less effect on DSB repair-proficient cells. PARP inhibition in recombination-deficient BRCA mutants is synthetic lethal (32,33). To analyze whether PARP activation by Dbait and Pbait has similar consequence as PARP inhibition, we tested siDNA toxicity in various BRCA mutant cell lines. We used breast cancer cell lines HCC1937 (BRCA1^−^^/^^−^) and MDAMB231 (BRCA^+/+^) as controls and HeLa cell lines silenced or not for BRCA1 and BRCA2 (Figure 7). We found that Dbait and Pbait had toxic effects on BRCA mutant cell lines but not on wild-type controls (Figure 7). As expected, the Bait32C, which has no free ends or nick and does not activate PARP or DNA-PK, had no effect on survival of the BRCA^+/+^ or BRCA^−^^/^^−^ cells (Supplementary Figure S6). In BRCA mutant cells, treatment with 0.1 μM Pbait gave similar survival than treatment with 10 μM ABT-888 PARP inhibitor (Supplementary Figure S6C and D). Microscopy monitoring of living cells after siDNA treatment showed that >50% of the BRCA^−^^/^^−^ cells treated by Pbait32 or Dbait32Hc undergo apoptosis within 6 h after treatment, whereas this event is extremely rare in BRCA^+/+^ control cell lines (data not shown).

## DISCUSSION

It is difficult to identify which particular signaling proteins are activated in cells in response to a particular type of DNA damage. This is because of the multiplicity of different types of damage caused by irradiation and the modification of the damaged DNA through replication and repair. To overcome these experimental problems, we have recently developed a new class of small molecules (called siDNA) that mimic one kind of damage each and that are not degraded, replicated or repaired in the cell. In this work, we used two kinds of siDNA molecules: Dbait, which mimics a DSB, and Pbait, which mimics an SSB. Both Pbait and Dbait activate the PARP polymerase activity. They differ in that Pbait activates only PARP, whereas Dbait activates both PARP and the kinase DNA-PK. In mammalian cells, the main pathway for the repair of DNA double-strand breaks (DSB) is NHEJ that depends on DNA-PK; however, when NHEJ is impaired, an alternative or back-up NHEJ (B-NHEJ) pathway dependent of PARP operates (34). Possibly, accessory proteins control a hierarchy in which DNA-PK-dependent regular NHEJ repair is privileged over PARP-dependent B-NHEJ (4,35). Our findings suggest that in the cell, the DNA-PK/Ku70/Ku80 complex and PARP can be equally recruited at the double-strand DNA ends of 32-bp-long Dbait molecules, the simplest structures that can mimic DSB. It is unlikely that both enzymes bind simultaneously to such small molecule. However, we cannot completely exclude this possibility, as recent structural analysis has revealed the formation of complex with DNA-PK and PARP on 60- or 54-bp-long DNA (36). Most DNA-damaging treatments cause many more other forms of lesions (SSB, base damages and so forth) than DSB, and these lesions presumably compete for PARP-1; this is consistent with PARP-1 not being the principal actor in the repair of DSB despite its higher affinity for DNA ends than Ku (37). In cells treated with Dbait, there are molecules mimicking ‘DSB’ and no other forms of DNA lesion, allowing the two pathways to be efficiently activated in the same cell: this was demonstrated by most of the cells displaying H2AX phosphorylation by DNA-PK also having substantial protein PARylation.

The first event observed after Pbait treatment is the formation of PAR foci. PARP-1 is one of the first proteins to recognize damaged DNA, and its interaction with DNA lesions triggers the PARylation of a variety of proteins, with PARP-1 itself being the main PAR acceptor (38). After DNA damage, the modification of PARP is estimated to represent 90% of the total PAR synthesis. There is evidence that PARylation may affect the chromatin structure to facilitate DNA repair processes. Interestingly, pre-extraction of soluble compounds from cells before PAR detection revealed that few chromatin components are PARylated after siDNA treatments. PAR acts not only as covalent protein modifications but also as protein-binding matrices (39)*.* This property could explain the formation of foci by XRCC1, PCNA and PARP that co-localize with PAR. As all these proteins are precipitated by pull-down with siDNA, it is likely that PARylated complexes built on the PAR (itself synthesized by the PARP bound to the siDNA) form aggregates. It has been suggested that Ku80 and DNA-PK bind to PAR (40). However, in contrast to PARP1, XRCC1 and PCNA, they did not form foci in Dbait32H-treated cells; therefore, it is unlikely that in our conditions they were recruited on the PARylated complexes. The lack of association of Ku and DNA-PK with PAR induced by Pbait32 is an interesting observation and suggests that the detection of the affinity of various proteins to PAR or PARylated PARP-1 (41,42) on damaged DNA should be re-examined. Alternatively, the polymers formed in response to Pbait may be different (in length or complexity) from those formed in response to chromatin DNA damage.

PARylation is transient, and the polymer is quickly degraded by PARG enzymes [poly(ADP-ribose) glycohydrolases] and poly (ADP-ribose) hydrolase 3 (ARH3) activities. The persistence of the PAR signal in cells treated with siDNA suggests that in these experiments, the siDNA remained in the cells for at least 2 days. NAD continued to be consumed during this period, ruling out the possibility that PAR persisted because of defective PARG and ARH3. The continuous consumption of NAD implies that PARP dissociates from the siDNA, the polymer is degraded and the native PARP re-binds the Dbait and synthesizes new polymers as previously proposed. However, this consumption, represent an increase of 50% in Dbait-treated cells as compared with untreated cells. The transitory NAD depletion was probably not the mechanism of cell death in BRCA^−^^/^^−^ cells, as similar depletion in BRCA^+/+^ cells did not affect proliferation and survival.

How Dbait and Pbait inhibit DNA repair? Here, we demonstrate that the general phosphorylation and PARylation after siDNA treatment prevents the recruitment of DNA repair proteins at the damaged locus on chromosomes. The SSB repair proteins associating with Dbait formed foci, and it is likely that they are trapped in these structures and consequently cannot move to, and contribute to the repair of the damaged chromosomal DNA. In contrast, the DSB repair proteins do not present such specific aggregation away from chromatin. The enzymes MRN and 53BP1 bind to damage independently of PARP and DNA-PK; hence, any inhibition of their recruitment is not likely to be a consequence of the trapping of the signaling enzymes on the siDNA. These proteins were not pulled-down with Dbait32Hc or Pbait32. The chromatin in cells treated with siDNA is extensively modified, as revealed by histone H2AX phosphorylation. It has long been known that all core histones are targets for phosphorylation after DNA damage (43). The resulting higher-order chromatin structure may be essential for facilitating the access of factors required for repairing DNA damage. After siDNA treatment, phosphorylated H2AX spread along all the chromosomes, and the organization of repair foci was impaired even after the localized accumulation of damage induced by laser. This inhibition may be due to a diffuse recruitment of the repair proteins over all the modified chromatin, which would considerably decrease the probability of a repair protein being at the damage site with all its partners. Also, we cannot exclude the possibility that the unscheduled phosphorylation of most of the repair proteins involved prevents appropriate organization of the repair process by modifying the interactions both between proteins and with the DNA.

Recently, Patel *et al.* (44) demonstrated that inactivation of NHEJ suppresses lethality induced by PARP inhibitors in HR mutants. They conclude that NHEJ activity is responsible of BRCA mutant cell death after PARP inhibition. According to this hypothesis, we would expect Dbait which inhibit both PARP and DNA-PK pathways to be not toxic in BRCA mutants. In contrast, we see an accumulation of unrepaired chromosomes and a decrease of survival after Dbait treatment in these cells. The fact that BRCA mutant shows higher sensitivity to Dbait than to Pbait, suggests that DNA-PK activation by Dbait is deleterious for survival. It is unclear whether the toxicity of Dbait in BRCA mutants is due to the inhibition of several DNA repair pathways (beside the PARP-dependent pathway also inhibited by Pbait) or is the consequence of cellular change induced by DNA-PK activation. In fact, the main differences between repair inhibitors (or siRNA) used by the group of Patel and Dbait is that the former acts on only one repair enzyme of the targeted pathway, whereas our molecules prevent binding at the damage site of almost all the repair enzymes we tested: proteins involved in NHEJ and HR such as (Ku70, DNA-PK, RAD51, MRE11, NBS1, 53BP1) (23,24) and SSBR proteins (PARP, XRCC1, PCNA) (this work) and possibly others as well. This multi-pathways inhibition would prevent alternative routes to restore repair.

Pbait and Dbait act as PARP inhibitors and are lethal in cells deficient in HR. Clinical applications of PARP inhibitors have attracted much attention during the past 10 years: ∼65 clinical trials testing eight different PARP inhibitors are currently in progress or have already been completed in various part of the world. Although PARP inhibitors have unprecedented therapeutic potential for the treatment of cancers, there is accumulating evidence that tumor resistance to these drugs develops in both preclinical and clinical settings (45). For example, tumors resistant to PARP inhibitors often show a reversion of the BRCA mutation or a defect in PARP expression. Moreover, a subset of BRCA1-deficient breast cancers had lost 53BP1 protein expression, resulting in the HR repair pathway being restored in these cells (46). Dbait is a repair pathway inhibitor acting on SSB repair as well as both NHEJ and HR. Its use could overcome the requirement of this therapeutic approach for a major defect in homologous recombination in tumors with high-genetic instability.

## SUPPLEMENTARY DATA

Supplementary Data are available at NAR Online: Supplementary Figures 1–6.

## FUNDING

Funding for open access charge: Institut Curie; Centre National de la Recherche; Institut National de la Sante et de la Recherche Medicale; Museum National d’Histoire Naturelle; CEE [STREP 28892 to Bioemergence]; Association Nationale de la Recherche et de la Technologie [743/2009 to A.C.].

*Conflict of interest statement*. A.C. is the recipient of a PhD fellowship co-financed by DNA Therapeutics. C.B. and M.Q. are employees of DNA Therapeutics. M.D. and J.-S.S. are cofounders of DNA Therapeutics.

## Supplementary Material

Supplementary Data
