# Supplementary material for: Inhibition of DNA damage repair by artificial activation of PARP with siDNA
Source: Nucleic Acids Res. 2013 Jun 12;41(15):7344–55. doi: 10.1093/nar/gkt522 (PMC3753643; doi:10.1093/nar/gkt522)
Supplement: Supplementary Data [file supp_41_15_7344__index.html]

Inhibition of DNA damage repair by artificial activation of PARP with siDNA — Inhibition of DNA damage repair by artificial activation of PARP with siDNA — Supplementary Data 

# Inhibition of DNA damage repair by artificial activation of PARP with siDNA

## Supplementary Data

files

**Files in this Data Supplement:**

- Supplementary Data - pdf file
