# Supplementary material for: Inhibition of DNA damage repair by artificial activation of PARP with siDNA
Source: Nucleic Acids Res. 2013 Jun 12;41(15):7344–55. doi: 10.1093/nar/gkt522 (PMC3753643; doi:10.1093/nar/gkt522)
Supplement: Supplementary Data [file supp_gkt522_nar-00901-d-2013-File009.pdf]

NAR-00901-D-2013: "INHIBITION OF DNA DAMAGE REPAIR BY ARTIFICIAL ACTIVATION OF PARP WITH SIDNA"

SUPPLEMENTARY FIGURES AND LEGENDS.

Supplementary Figures 1-6.

Figure S1.
Bait32C, a siDNA dumbbell molecule, do not induce PARP and DNA-PK activation.

Figure S2.
Kinetic of NAD consumption induced by Dbait and Pbait molecules.

Figure S3.
PARylation induced by Dbait and Pbait in several cell types.

Figure S4.
PARylation induced by Dbait molecules is not DNA-PK dependent.

Figure S5.
Pbait prevent the relocalisation of PARP but not NBS1 and MRE11 repair proteins after irradiation.

Figure S6.
SiDNA act as PARP inhibitor and induce synthetic lethal effect.

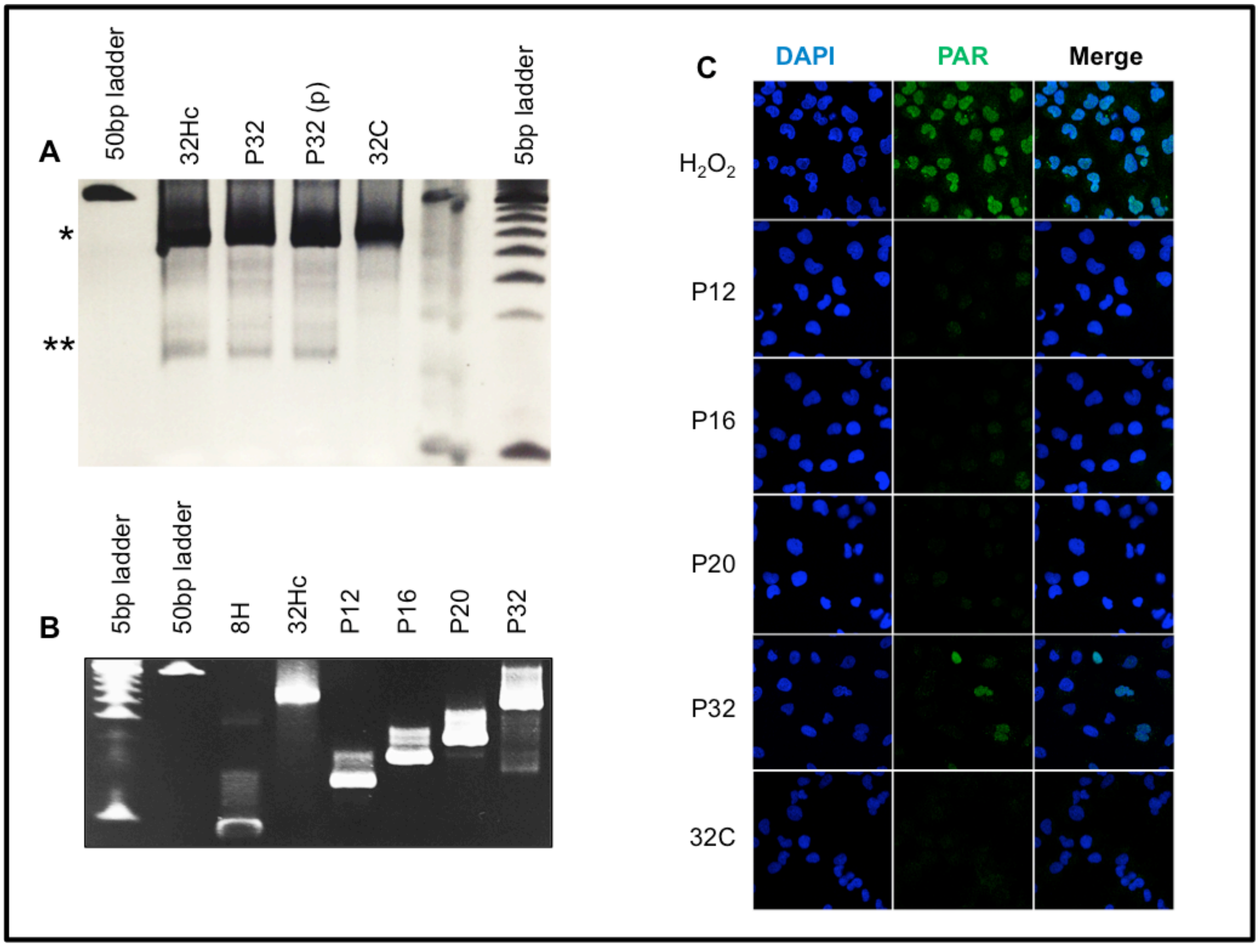

Figure S1. Bait32C, a siDNA dumbbell molecule, do not induce PARP and DNA-PK activation. (A) Construction of dumbbell molecules: Final (Bait32C noted 32C) and intermediate products (Pbait32(p) noted P32(p)) were analyzed by electrophoresis. 1.2nmol of Pbait32 molecules were diluted in 100µl of 1X ligation buffer (New England Biolabs, Ipswich, USA); heated 5 minutes at 70°C before adding T4 kinase (New England Biolabs, Ipswich, USA) (40 units) and incubated 60 minutes at 37°C (P32(p) molecules). T4 ligase (New England Biolabs, Ipswich, USA) (5 units) was added and the mix was incubated at 16°C during 1h, and then heated 10 minutes at 65°C to inactivate the enzyme (32C molecules). Ligation products were analyzed on a denaturing 15% acrylamide + 7M urea gel (in 10ml: 3,75ml acrylamide-bisacrylamide (40%), 4.2g Urea, 1ml TBE 10X, 100µl APS (10%), 7µl Temed, qsp H_20) after 1h migration at 200V. Due to the high stability of the molecules (Melting Temperature (MT) > 95°C) native forms (*) and denatured forms (**) were observed for all samples except Bait32C, which was not denatured after ligation. (B) Analysis of various siDNA used in this study in a non-denaturing 15% acrylamide gel. (C) Immunofluorescence of PAR (green) in MRC5 cells transfected with Pbait12 (P12), Pbait16 (P16), Pbait20 (P20), Pbait32 (P32) and Bait32C (32C) molecules or treated with H_2O_2 (500µM).

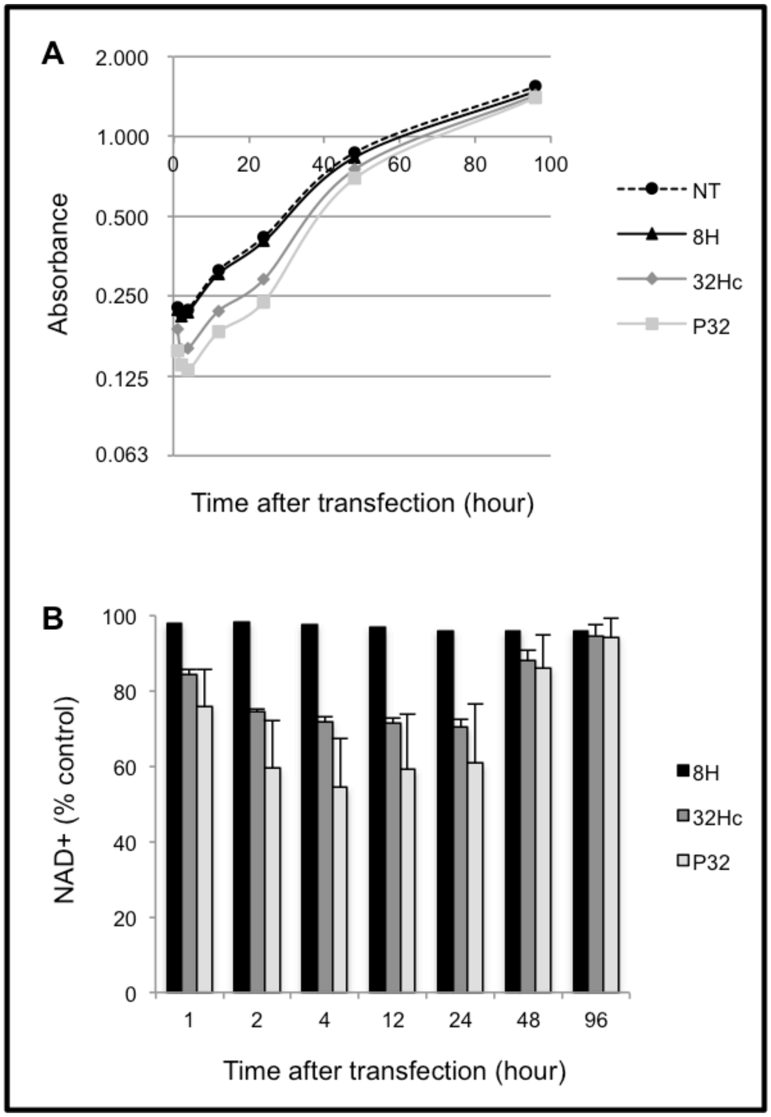

Figure S2. Kinetic of NAD consumption induced by Dbait and Pbait molecules. Kinetic of NAD consumption induced by Pbait32 (light gray), Dbait32Hc (dark gray) and Dbait8H in MRC5 cells during 4 days. All experiments were done in triplicate. Absorbance measurement (A) and calculated ratio (B) in treated and untreated samples. NAD quantification was determined by the CCKit8 test (Dojindo, Maryland, USA). Cells grown in 24-well plate were treated with 0.2µg siDNA molecules for 5h in 200µl complete medium. WST-8 [2 - (2-methoxy-4-nitrophenyl) - 3 - (4-nitrophenyl) - 5 - (2,4-disulfophenyl) - 2H - tetrazolium, monosodium salt] was added and the water-soluble formazan dye produced by reduction of WST-8 during NAD consumption was monitored by absorbance at 450nm. Samples were measured at different times during 4 days. All experiments were done in triplicate. Since cells growth was not affected by siDNA treatment, the difference observed during the 24 hours is probably due to the induction of PARP activity in the treated cells.

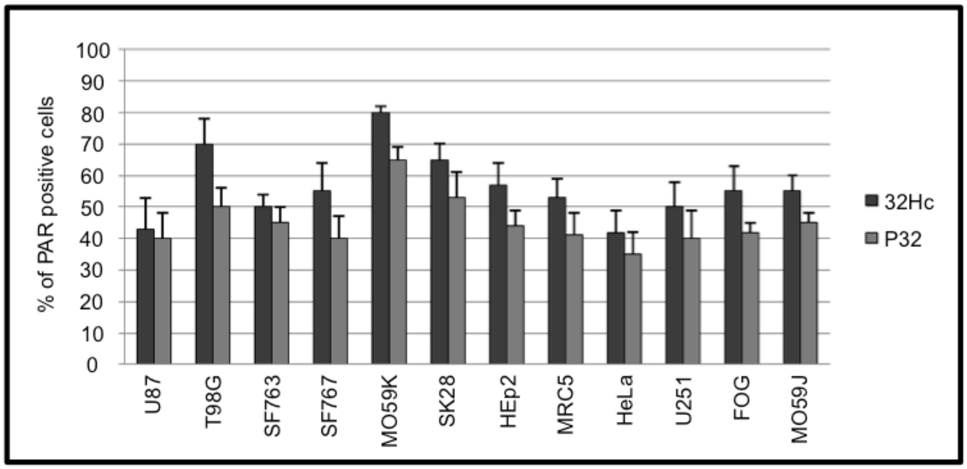

Figure S3. PARylation induced by Dbait and Pbait in several cell types. Quantification of PAR positive cells transfected with Dbait32Hc (dark gray) and Pbait32 (light gray), in glioblastoma (U87, T98G, SF763, SF767, MO59K, MO59J, U251, FOG), melanoma (SK28) and cervix/larynx cancer (HeLa and HEp2) cell lines. Percent of cells is the mean of 100 cells counted.

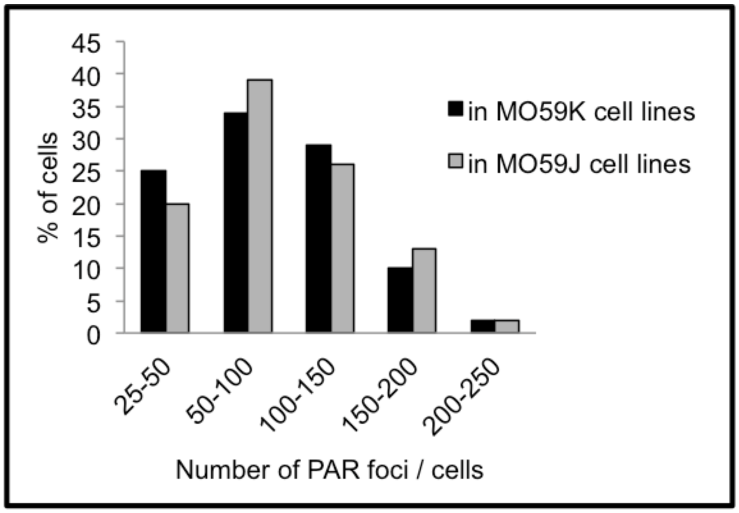

Figure S4. PARylation induced by Dbait molecules is not DNA-PK dependent. Distribution of PAR in MO59J (DNA-PK deficient) cells (gray) and MO59K (DNA-PK wild-type) cells (black) treated with Dbait32Hc molecules. Percent of cells is the mean of 100 cells counted.

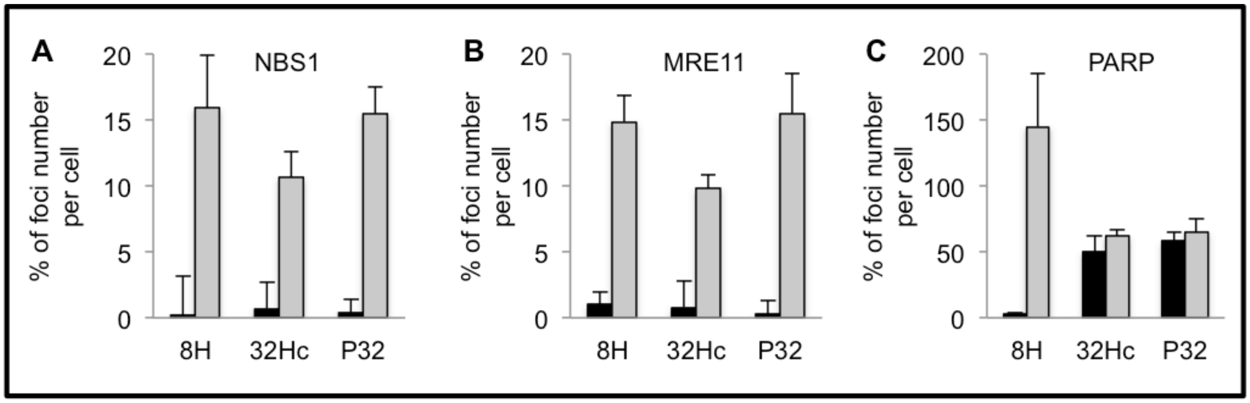

Figure S5. Pbait prevent the relocalisation of PARP but not NBS1 and MRE11 repair proteins after irradiation. Numbers of foci of NBS1 (A), MRE11 (B) and PARP (C) proteins formed in cells transfected with Pbait32, Dbait32Hc or Dbait8H and irradiated (gray) or not (black) with 10Gy.

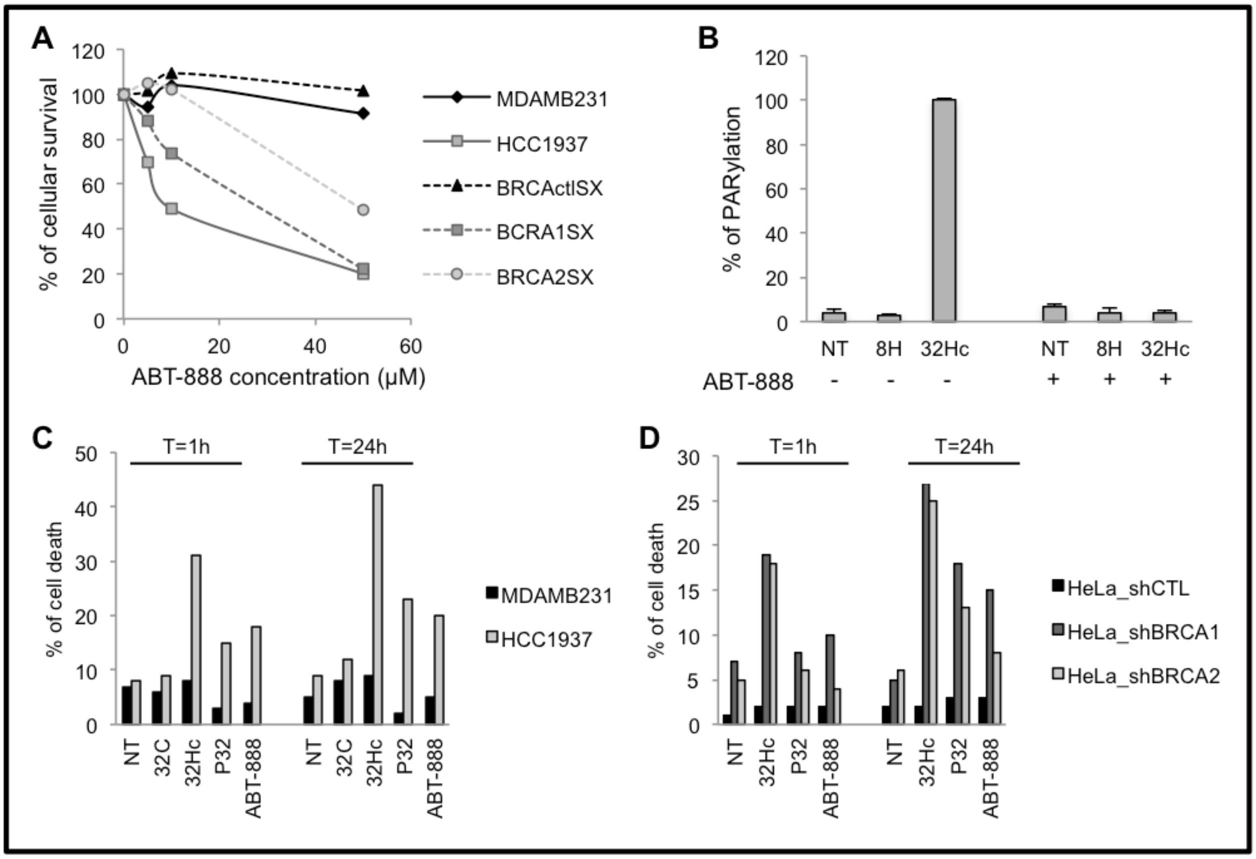

Figure S6. SiDNA act as PARP inhibitor and induce a synthetic lethal effect in BRCA deficient cell lines. (A) Cell sensitivity to PARP inhibitors (ABT-888). Cells were seeded in 24-well plates at concentration of 250, 500 and 1000 cells per well. Triplicate wells were processed for each experimental point. ABT-888 treatments (5, 10 and 50µM) were performed the day following seeding. Cells were then allowed to grow for nine days, fixed for 20 minutes in 4% formaldehyde/PBS, permeabilized with 0.5% Triton X-100 for 10 minutes and stained with To-PRO 3 (Invitrogen, Grand Island, USA) diluted 1/800 for 10 minutes. After staining, the 24-well plates were analyzed and quantified by Odyssey (LI-COR Biotechnology, Bad Homburg, Germany). Analyses were performed in breast cancer cell lines (solid lines: MDA-MB-231 ($BRCA^{+/+}$), black; HCC1937 ($BRCA1^{-/-}$), gray), and in HeLa cells (dotted lines: HeLa_shCTL, black; HeLa_shBRCA1, dark gray; HeLa_shBRCA2, gray). (B) PARylation activities were measured in nuclear extracts from MRC5 cells. PARP activation was estimated by measuring H1 histone parylation after addition of 10µM of PARP inhibitor (ABT-888) before siDNA (0.2µM) treatment. Values were normalized to the maximal Dbait32Hc activity for each condition. Reported values represent the mean value and standard deviation of at least three independent experiments. The Dbait8H molecules were used as negative control. (C, D) Cellular survival was monitored in Bait32C (32C); Dbait32Hc (32Hc); Pbait32 (P32) or ABT-888 treated cells by Trypan blue cell counting. Analyses were performed in breast cancer cell lines (panels C: MDA-MB-231 ($BRCA^{+/+}$), black; HCC1937 ($BRCA1^{-/-}$), gray), and in HeLa cells (panels D: HeLa, black; HeLa_shBRCA1, dark gray; HeLa_shBRCA2, gray).
